# Immune Dysfunction in Tourette Syndrome

**DOI:** 10.3233/BEN-120295

**Published:** 2013

**Authors:** Ishraga Elamin, Mark J. Edwards, Davide Martino

**Affiliations:** ^1^Sobell Department of Motor NeuroscienceInstitute of NeurologyUniversity College LondonLondonUK; ^2^Centre for Neuroscience and TraumaQueen Mary University LondonBarts and the London School of Medicine and DentistryLondonUK; ^3^Queen Elizabeth Hospital WoolwichSouth London NHS TrustLondonUK

**Keywords:** Tourette syndrome, immunity, T cells, antibodies, neuroinflammation, allergy

## Abstract

The association between immunity and neurodevelopmental disorders has been extensively investigated in autism, suggesting a potential involvement of both cellular and humoral immunity in the establishment of synaptic connectivity modulation during development. A similar link has been proposed also for Tourette syndrome (TS), a complex, multifactorial disorder, in which the interplay between genetic, environmental, hormonal and immunological factors might be relevant. Lymphocyte subpopulation analysis in TS suggests a possible systemic activation of several T- and B-cell subtypes, whereas the observed decreased numbers of T regulatory lymphocytes might predispose to autoimmunity. Genes related to both cell- and antibody-mediated immune responses may be over-expressed at specific ages in youngsters with TS. Data from cytokine measurements and transcriptomics profiles in TS patients are coherent with the systemic immune activation detected by studies on lymphocyte subpopulations. Moreover, TS patients have exhibited IgG3 and IgA dysgammaglobulinemia, which might predispose to recurrent infections and autoimmunity. To date, the association between TS and autoantibodies has not been demonstrated. Interestingly, however, there is a higher degree of maternal family history of autoimmune diseases among TS patients. Finally, TS patients could be prone to allergic illnesses (asthma, atopic dermatitis, rhinitis, conjunctivitis), but more work is needed in this area.

## 1. Introduction

The involvement of the immune system in Tourette syndrome (TS) is a relatively recent area of investigation, originally driven by the description of clinical series of ‘post-infectious’ Tourette-like syndrome (or tourettism). Pathogens advocated to be responsible of this form of tourettism include Group A ß-haemolytic streptococcus (GABHS) [1], *Mycoplasma pneumoniae* [2], *Borrelia burgdorferi* [3], and different viruses [4]. Beyond a potential link to infections, the involvement of immunity in the pathogenesis of several common neurodevelopmental disorders, particularly autism, is increasingly explored, suggesting a role of immune proteins and cells in the abnormal establishment and modulation of synaptic connections during development in these disorders [5–7]. This review will summarise the current knowledge on the involvement of immune responses in TS and synthesize existing viewpoints on the interaction between immune and nervous systems in the pathobiology of TS.

## 2. Immune cell subpopulations

Figure 1 provides a rapid summary of the organisation of normal human immune response. Human leukocyte antigen (HLA) typing is the most widely used immunophenotype marker in human pathology. Only limited information in respect to HLA subtypes has been collected over the years from patients with TS, which has been overall inconsistent [8,9]. Schoenian et al. [10] genotyped the HLA-DRB subtypes, known to be engaged in antigen presentation mechanisms in 83 TS trios, formed by the affected index child and both parents, and tested the association with the diagnosis using a non-parametric method, the transmission disequilibrium test. The findings of this study are not in favour of an association between the HLA-DRB lo-

ISSN 0953-4180/13/$27.50 © 2013 – IOS Press and the authors. All rights reserved

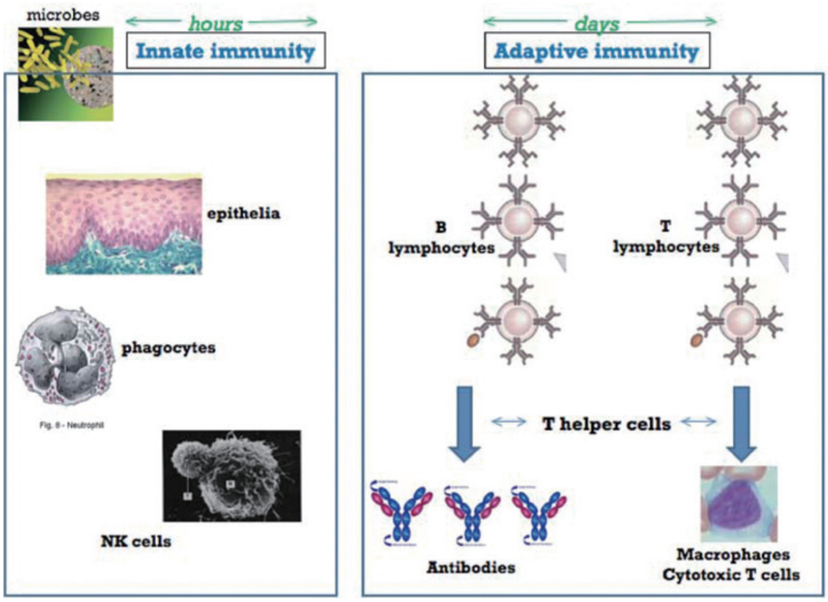

Fig. 1. The immune system has two functional arms, the innate and the adaptive arms, which cooperate to protect the host against infections. They differ in their way of recognizing pathogens: the innate immune system recognizes a small number of pathogen-associated molecular patterns through a small number of different receptors, and represents the first line of inflammatory defense against pathogens, whereas the adaptive recognizes a wide multitude of antigens, presented by specialized antigen-presenting cells, thus building up an antigen-specific response in a more delayed fashion. Phagocytes and natural killer cells are the principal actors of innate immunity, and are specialized in killing intracellular pathogens, whereas T and B lymphocytes are the main ones in adaptive immunity, and support cell-mediated and antibody-mediated effector responses. Among T lymphocytes, T helper lymphocytes (or CD4+) are the 'orchestra conductors', modulating both cell-mediated activity of macrophages and cytotoxic T lymphocytes and antibody production by plasma cells which originate from B lymphocytes. In immune-mediated diseases, either cellular or humoral responses often predominate one on the other, and this is relevant also in therapeutic terms.

cus and TS. However, these studies might have lacked sufficient power to detect an association between HLA genotype distribution and a particular subgroup of TS patients, and a wide number of other polymorphic variants in genes connected to immune responses, such as polymorphisms in the T cell receptor loci, have never been explored in this condition to date.

The general features of the immune response in a single individual at a definite point in time can be also expressed by the quantitative distribution of the various immune cell subpopulations in the peripheral blood, as well as by their functional state. The count of immune cell subpopulations is performed using fluorescence activated cell sorting (FACS). Probably the most intriguing finding obtained applying this technique to peripheral blood mononuclear cells of TS patients is the 62% decrease in percentage of *naïve* CD4+CD25+ T cells (i.e., Tregs) on the total number of *naïve* CD4+ T cells (i.e., T helper cells or Th) in 37 children with moderate-to-severe TS and/or obsessive-compulsive disorder (OCD), compared to 9 healthy subjects [11]. Th cells represent the main regulatory element of adaptive immune responses. Tregs are a subpopulation of Th cells which is involved in the basic mechanisms of immune tolerance towards self antigens, thus protecting against autoimmune insults. In fact, reduced numbers of Tregs are detected in autoimmune conditions like lupus erythematosus, rheumatoid arthritis, type 1 diabetes, and multiple sclerosis, whereas increased levels of Tregs are seen in hepatitis B and C viruses and cytomegalovirus infections, and in several types of cancer [12]. In TS and/or OCD children from this study [11], Tregs tended to decrease particularly during symptom exacerbations, although this did not reach statistical significance.

In an exploratory pilot study by Moller et al. [13], 20 adults with TS were compared to 20 age-matched healthy subjects, showing a 65–75% higher percentage of CD69+ B cells, and an approximately 65% higher percentage of CD4+CD95+ and CD8+CD95+ T cells in TS patients compared to control subjects. CD69 is a general marker of B cell activation; CD95 (also known as Fas) is involved in the induction of apoptosis of T cells: it is possible that its expression is up-regulated as a compensatory mechanism for excessive T cell activation in these patients. The results of this cross-sectional study are in favour of a generalized over-activation of lymphocytes in TS, although they do not provide sufficient information on the cause-effect relationship between immune activation and abnormal behaviours.

Additional, although indirect, support in favour of a widespread activation of lymphocytes in TS patients came from the work of Hoekstra et al. [14], who observed that, compared to healthy subjects, patients with tics over-expressed immunoglobulin receptors on the surface of peripheral B cells. This finding potentially indicates a generalised functional activation of B cells in these patients, although the trigger to this overactivation remains unknown. Weisz et al. [15] also observed a significantly higher percentage of CD19+ B cells in the peripheral blood of a smaller TS patient population.

Overall, although limited in number, the available studies exploring the functional state of immune cells in TS through surface receptor typing (immunophenotyping) provide important preliminary evidence in favour of a broad over-activation of immune cells in these patients. The interpretation of these findings needs, however, to take into account the several limitations of these previous works. Definitely more work is needed on larger clinical samples in order to confirm the reported findings and attempt a characterization of a subgroup of TS patients in whom overactive immune responses are more striking. Indeed, in addition to tics, other factors might influence immune responses in these patients, among which age, medications (particularly those modulating dopamine receptors, which might also exert immunomodulatory effects), and the complex spectrum of behavioural co-morbidities of TS. Immunophenotyping analyses should be broadened to comprise other important functional subtypes, such as the different Th subtypes (Th1, Th2, Th3, Th17): the relative distribution of these different Th subtypes is very important for the general regulation of immune responses. Prospective assessment of a sufficient number of these patients would also prove useful to explore further the cause-effect relationship between neuropsychiatric symptoms and immune functioning. Finally, it is poorly understood whether these immunophenotype changes are related to environmental triggers, such as streptococcal or other infections, or stress.

## 3. Immune effector molecules

A series of peptides expressed and released by immune competent cells are crucial for the organisation of cell-mediated and antibody-mediated responses. Cytokines are released, often in cascade fashion, at inflammatory sites by all the main immune competent cell types. Within adaptive immune responses, different cytokines convey the regulatory effect of different T cell subtypes, some displaying pro-inflammatory and others anti-inflammatory properties. Chemokines exhibit chemoattractant properties to mediate the recruitment of lymphocytes towards inflammatory sites. Finally, adhesion molecules mediate the extravasation and entry of circulating lymphocytes into inflammatory sites. Circulating levels of these effector molecules may give information on the actual state of activation of immune-inflammatory responses.

A small number of cross-sectional studies, heterogeneous for sample size, clinical features, and exposure to psychotropic medications, have analysed serum levels of effector molecules in patients with tics and age-matched healthy subjects. Results have been overall inconsistent. The most noteworthy finding from these studies is the presence of higher circulating levels of interleukin-12 (IL-12), an activator of Th1 and Natural Killer (NK) cells, in patients with tics, which has been reported independently by two groups of authors [16,17]. The earlier of these studies [16] has, however, compared a mixed population of children with a tic disorder and/or OCD to healthy subjects, whereas the second [17] found raised IL-12 levels only in children with TS+OCD, but not in those with TS alone. However, Bos-Veneman et al. [18], in the largest to date cross-sectional study on cytokine levels in children with TS, found no difference in IL-12 levels between 66 patients and 71 healthy volunteers, and obsessive-compulsive symptom severity was inversely related to IL-12 serum levels in the patient group. Other effector molecules have been explored [16–18], leading to scattered findings of raised interleukin-2 (IL-2), tumour necrosis factor-$\alpha$ (TNF-$\alpha$), and soluble adhesion molecules, which could not however be replicated.

Data from prospective longitudinal observations of cytokine levels and severity of neuropsychiatric symptoms are even more limited. Leckman et al. [16] observed an increase of a wide array of pro- and anti-inflammatory cytokines at symptom exacerbation compared to pre-exacerbation visits in a mixed group of children with tics and/or OCD. Statistical significance was, however, reached only for IL-12 and TNF-$\alpha$ levels, with a positive correlation between tic severity and TNF-$\alpha$ levels. These fluctuations could not be observed in children with the putatively ‘post-streptococcal’ Tourette-like illness named Pediatric Autoimmune Neuropsychiatric Disorders Associated with Streptococcal Infections (PANDAS) [16,19].

In respect to acute inflammatory markers, erythrocyte sedimentation rate and C-reactive protein (CRP) are not raised in TS patients, nor co-vary with tic severity [20,21]. Serum levels of neopterin, a pteridine produced by human monocytes/macrophages especially during cell-mediated immune responses, was found raised in two independent cross-sectional observations of TS patients compared to healthy subjects, although there was no significant difference in neopterin serum levels between pre-exacerbation and exacerbation [21, 22]. Interestingly, neopterin may be raised in several autoimmune diseases, including systemic lupus erythematosus, rheumatoid arthritis, and multiple sclerosis. Despite the lack of correlation with symptom severity, the association between this marker and TS has been confirmed in two independent cohorts [21,22].

## 4. Gene expression profiling of immune cells

Microarray profiling of genome-wide expression changes (transcriptomics) allows the measurement of the activity of dysregulated molecular pathways within peripheral immune cells, allowing for the identification of disease-specific gene expression fingerprints. Fourteen NK cell genes were over-expressed in 10 of 16 familial cases of TS compared to 16 control subjects [23] using these methods. A subsequent study from the same group [24] on 28 case and 30 control children/adolescents found that the expression of a number of genes and multiple pathways differed between TS and control subjects within different age strata (5 to 9 years, 10 to 12 years, 13 to 16 years). Particularly, age-related expression changes specific of TS patients were observed for genes involved in viral response, including interferon processing and B cell immunoglobulin synthesis, and cell-mediated cytotoxicity mechanisms involving NK and CD8+ T cells. This finding might suggest an abnormal immune recognition of pathogens in TS patients, which might indirectly account also for an increased synthesis of cytokines in these patients. However, the age-dependent change in expression of immune regulatory proteins [24], although intriguing, was observed across different age strata within a cross-sectional analysis of a clinical sample of TS patients. Truly prospective observations are necessary to confirm this finding.

Although there is increasing evidence of abnormally regulated immune responses in TS, it is likely that these changes differ in size across the whole population of patients, with a subgroup of TS patients more 'immunologically impaired' than others. The characterization of this subgroup will be possible only through better designed longitudinal studies of sufficiently large clinical samples. Peptides involved in the immune regulatory mechanisms might also be engaged in neural development, and further work is needed to identify which of these molecules are involved in the pathogenesis of TS and whether they exert harmful or beneficial effects over the maturation of specific brain networks in patients with neurodevelopmental disorders. More work is also needed to clarify whether TS patients develop abnormal immune responses against viral, bacterial or parasitic pathogens, which might contribute in some way to tic onset and/or exacerbation.

## 5. Neuroinflammation

It is currently uncertain whether TS patients develop relevant neuroinflammatory changes in pathologically relevant regions. This lack of information is obviously due to the ethically limited availability of pathological and CSF specimens from these patients.

Microarray transcriptomics studies analysed very small series of brain specimens ($n = 3$ and $n = 4$ in two independent studies) from adults with TS and neurologically healthy adults. Morer et al. [25] showed a 2.3-fold elevation of IL-2 gene transcript expression in the basal ganglia of TS brains compared to control brains. In line with this finding, Hong et al. [26] reported a 2.9-fold increase of IL-2 receptor ß gene transcript expression in *post mortem* putamen of TS patients, suggesting increased activation of the IL-2 system, a major T-cell-derived growth factor, in the basal ganglia of TS patients. Morer et al. [25] also found a 6.5-fold elevated expression of monocyte chemotactic factor-1 (MCP-1) in the same brain specimens. MCP-1 has interesting functional properties: a) it is a chemokine that may increase blood-brain barrier permeability, allowing the infiltration of leukocytes and antibodies in the nervous tissue; b) it is involved in the differentiation of neural stem cells into neurones, astrocytes and oligodendrocytes [27], which seems interesting, in the light of an observed abnormal distribution of interneurons in the basal ganglia of TS patients.

The search of neuroinflammatory changes in the CSF is likewise limited in TS. Wenzel et al. analysed the CSF of 21 TS patients of age ranging between 9 and 51 [28]. In spite of the absence of noteworthy blood-brain barrier leakage in the vast majority of these patients, isoelectric focusing analyses of oligoclonal bands found positive ($\geqslant$ 4 bands) or borderline (2–3 bands) intrathecal IgG production in 8 of the 21 patients (38%); other two patients displayed a mirrored oligoclonal band pattern. A control group specifically collected *ad hoc* for this study was not available, but a standard CSF examination on a historical cohort of 99 healthy subjects in the same laboratory detected CSF oligoclonal bands only in 3%. Therefore, intrathecal antibody synthesis might be occurring in a subgroup of TS patients.

Finally, it is useful to point out that the role of microglia deserves further exploration in this condition. Apart from its action as antigen-presenting cell in the brain, microglia may have a role in the maintenance of synaptic integrity and of the activity of specific pathways [29]. A recent seminal work has shown that a mutation of the *Hoxb8* gene inducing a loss of function in a microglia lineage of hematopoietic origin in mice led to the development of a phenotype consisting in compulsive hair removal, which was homologous to OCD spectrum behaviours in humans [30].

## 6. Immunoglobulins

Recent work has shown that patients with TS display an abnormal profile of immunoglobulin production, possibly another aspect of the dysregulated immune activity in this condition. Plasma levels of all immunoglobulin subtypes (IgG1, IgG2, IgG3, IgG4, IgM, IgA, and IgE) were measured in two independent cohorts (53 Dutch and 21 US patients) of youngsters with TS and compared to equals numbers of healthy age- and centre-matched volunteers [31]. Both TS cohorts displayed significantly lower IgG3 levels than controls; there was also a trend for reduced levels of IgM, whereas other immunoglobulin levels did not differ between TS and healthy in both cohorts.

In line with earlier work [32], Kawikova et al. compared total levels of IgG, IgM and IgA between 24 children/adolescents with TS/OCD, mostly fulfilling criteria for PANDAS, and 22 healthy subjects, showing lower levels of total IgA in patients [33]. Additional work is needed to confirm whether IgA dysgammaglobulinemia can be an immunological marker for this putatively 'post-streptococcal' tourettism, and to explain how this relates to the pathogenesis of this controversial entity.

If confirmed, IgG3 and IgA dysgammaglobulinemia may suggest an interesting link between exposure to infections and abnormal immune responses in TS. Reduced IgG3 synthesis may predispose TS patients to several types of infections, given that one of its main roles is to facilitate neutralisation and killing of microbes by activation of the complement cascade and activation of innate immune cells, such as NK cells [34]. Reduced IgA synthesis, if observed also at the level of IgA secreted by mucosae, might make mucosal surfaces more vulnerable to microbial colonisation; moreover, reduced IgA synthesis might disinhibit cell-mediated immune responses and favour autoimmunity, as suggested by the high rate of autoimmune disorders in patients with IgA deficiency [33].

## 7. Autoimmunity

As detailed above, TS patients might be indirectly prone to autoimmunity, due to loss of tolerance to the self (decreased number of Tregs) or disinhibition of autoreactive lymphocytes (decreased IgA synthesis). Whereas cell-mediated autoimmunity is virtually unexplored in TS, autoantibody-mediated autoimmunity has been extensively investigated, but with conflicting results [36].

The most recent views on autoantibody-mediated pathogenicity within the neural tissue suggest that pathogenic autoantibodies bind surface (membrane-bound) antigens at the level of specific sites (or epitopes) that bear a particular tridimensional conformation *in vivo*. One important caveat of many previous works that used immunological methods, such as Western blotting or enzyme-linked immunosorbent assay (ELISA), is that they identified antibody binding to tissue antigens following denaturing processes that dissolved the complex tridimensional structure of antigens. In this way, antibodies from patients' specimens bound to 'artificial' linear epitopes which might not have been really exposed to antibodies in vivo, thus displaying non-specific and clinically irrelevant antibody reactivity [35]. Truly pathogenic autoantibodies, instead, may be identified using live cell systems, such as neuroblastoma or transfected cell lines like HEK cells. Earlier studies [36,37] demonstrated that TS and PANDAS sera contained autoantibodies binding to the cell surface of neuroblastoma cell lines or rat striatal neurons. These authors scored visually this binding using immunofluorescence. Conversely, when IgG cells surface binding to live differentiated neurons and to a non-neuronal control cell line was quantitatively measured with more accuracy using flow cytometry, no significant difference in IgG cell surface binding was detected between sera from 12 patients with PANDAS, 11 with TS, and two groups of control subjects [38]. The small sample size in this more recent flow cytometry work does not allow, however, to draw firm conclusions on the true frequency of autoantibodies in TS.

Regardless of their frequency, autoantibodies targeting neural tissue would be an important area for future investigations in TS only if functionally relevant and potentially contributing to the pathogenesis of tics. The target of anti-neural antibodies in this condition is, in any case, still undetermined. Given that patients with PANDAS are phenomenologically similar to TS and have been hypothesized to be associated with an antibody-mediated mechanism [1,37], it is not surprising that autoantibody reactivity detected in children with post-streptococcal neuropsychiatric disorders, e.g. Sydenham's chorea and PANDAS, has been explored also in the general population of TS patients. These targets include the membrane isoform of glycolytic enzymes, particularly pyruvate kinase M1, which was identified as a self-antigen in TS by two independent groups [39,40]. Although their function on neuronal membranes is still not completely clear, these glycolytic enzymes might be involved in energy metabolism and ion channels support [41]. Altogether, anti-neuronal glycolytic enzyme antibodies have been reported in 20–25% of children with TS from two large cohorts, although they were not associated with basal ganglia volumes or tic severity when patients were followed-up prospectively [20,42]. Other groups, however, did not confirm these findings, and their true pathogenic potential has not been demonstrated yet [43,44]. Other putative self-antigens, previously identified in Sydenham's chorea, such as lysoganglioside GM1 and tubulin [45], have not been adequately explored in typical TS, although one study did not identify anti-lysoganglioside GM1 antibodies in 24 youngsters with TS/OCD (80% of whom fulfilled PANDAS criteria) [33].

Additional criteria to support pathogenicity of anti-neural antibodies in TS include their presence in the brain, the demonstration of a model with face validity for TS characterized by antibody-mediated mechanisms in experimental animals, and the therapeutic potential of immune-modifying treatments that remove autoantibodies from patients [46]. The presence of immune complexes in pathological brain specimens from TS patients has never been shown, but *post mortem* material from these patients is exceedingly rare. Stereotactic striatal infusion of patients' antibodies in rodents gave inconsistent results [47,48]. whereas active immunisation with rheumatogenic streptococcal strains led to the development of rodent models with an antibody-mediated behavioural disorder with some face validity also for tic disorders [49,50]. In one of these studies, a pathogenic role for anti-human D1/D2 dopamine receptor antibodies has been postulated, but their presence in a subgroup of patients with TS is still unknown and the object of ongoing research [50]. Immune-modifying treatments like plasma exchange and intravenous immunoglobulins are not beneficial in unselected populations of TS patients refractory to less invasive therapies [51], but might be effective in reducing obsessive-compulsive symptoms and tics in youngsters fulfilling criteria for PANDAS [52]. However, the clinical definition of PANDAS is undergoing major revision and therefore it is premature to conclude that immune-modifying treatments can be used in a particular subgroup of patients with tic disorders.

Do we have any additional evidence in support of an important role of autoimmunity in TS? Common markers detected in systemic autoimmune diseases, such as anti-nuclear antibodies, were not consistently found in TS [53,54]. Likewise, the detection of antiphospholipid antibodies in TS is likely to be a purely coincidental occurrence lacking any pathogenic relevance [55]. Probably the most intriguing direct finding in support of a predisposition for autoimmunity in children with tics and/or OCD is the observation of an increased rate of autoimmune diagnoses in their mothers. Murphy et al. reported that about 18% of mothers of children with these neuropsychiatric disorders were diagnosed with an autoimmune illness, a percentage that is substantially higher than the general prevalence of autoimmune diseases in women living in the US [56]. These authors also found that the frequency of self-report of autoimmune diseases was relatively higher in mothers of children with "likely PANDAS" versus "unlikely PANDAS". Interestingly, a higher familial risk of autoimmune diseases has been reported also in patients with autistic spectrum disorders, in whom 47.5% of patients have a family member with an autoimmune disease (approximately 5-fold more than control populations) [57]. These findings might suggest a genetic predisposition to the development of autoimmune mechanisms and/or a higher risk of prenatal damage caused by antibodies to fetal brain tissue during pregnancy, potentially affecting normal brain development. The existence of antibodies to fetal tissue produced by mothers of children with TS or OCD is unknown, but seems however very difficult to explore.

## 8. Allergy

Following an earlier work that detected an increased rate of allergic reactivity to a multitude of different al-

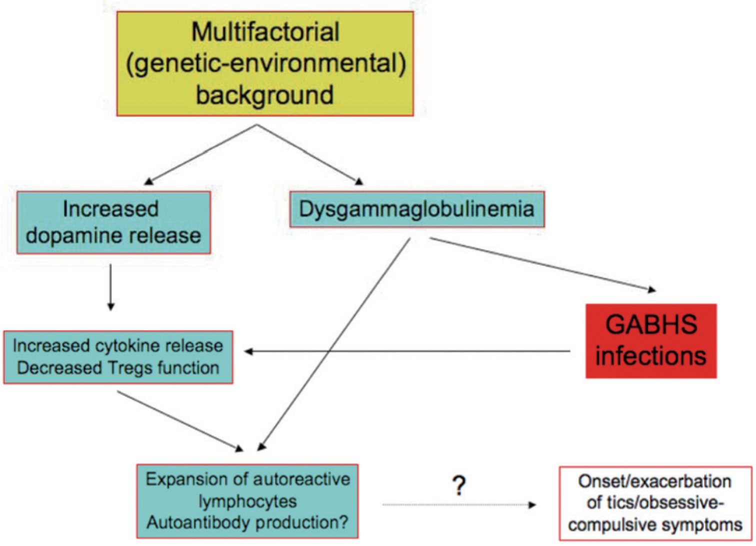

Fig. 2. This diagram summarises the mechanistic series of events that might lead to and sustain immune dysfunction in Tourette syndrome (TS). Enhanced cytokine release and reduced immune tolerance secondary to reduced function of T regulatory lymphocytes (Tregs) might be facilitated by the generalised hyper-dopaminergic state and possibly also by dysgammaglobulinemia. These immunological changes might subsequently promote autoimmunity. Group A beta-haemolytic streptococcal (GABHS) infections might be facilitated by the co-existing dysgammaglobulinemia of the host, and, at the same time, enhance immune activation, possibly exerting also a direct effect on autoimmunity in the presence of molecular mimicry between brain and microbial antigens.

lergens in the population of Taiwan [58], Chang and co-workers conducted a retrospective case-control study on this topic using a health insurance national database from the same country [59]. Comparing 845 children/adolescents with TS to 3378 control subjects of similar age, gender and level of urbanization, these authors found a higher risk for allergic rhinitis, asthma, dermatitis and conjunctivitis in TS patients, which was not confounded by age and co-morbidities. More research is needed to confirm the possible proneness of TS patients to allergic mechanisms.

## 9. Final commentary

The majority of the immunological changes reported in TS patients ideally need to be confirmed in larger population samples. Although available data suggest that the regulation of immune responses is abnormal in TS, it is still unknown whether such abnormalities are present at different degrees in all patients with TS, or, rather, immune dysfunction is characteristic of a specific subgroup within the heterogeneous spectrum of TS. Also, more research is necessary to understand whether immune dysfunction worsens tics and other abnormal behaviours and/or contributes to their pathogenesis, or, conversely, represents an epiphenomenon secondary to the same primary mechanisms accounting for tics and other dysfunctional behaviours. Figure 2 provides a mechanistic model to explain the development and persistence of immune dysfunction in TS.

One fundamental question remains the following: are infections important for the immune dysfunction observed in TS? In the Autoimmunity section, we summarized current hypotheses involving molecular mimicry between brain and GABHS to explain autoantibody production in post-streptococcal neuropsychiatric disorders, including PANDAS. The observed higher risk of children with tics and obsessive-compulsive symptoms of having contracted throat infections in the 3 months prior to neuropsychiatric onset suggests increased exposure of these patients to pathogens like GABHS, regardless of whether they fulfill the diagnostic criteria for PANDAS [60]. Also, Bombaci et al. elegantly showed that children with tics develop an antibody response towards a much broader set of streptococcal antigens compared to children with acute streptococcal pharyngitis and healthy children [61]. On one hand, it is therefore possible that children with TS are more exposed to this and possibly other pathogens, particularly before the first onset of tics and obsessive-compulsive symptoms. On the other hand, TS patients might constitutively develop hyperactive immune responses, which can be particularly enhanced by powerful immunogenic triggers, such as GABHS, thus exalting first-line inflammatory responses and/or cytokine release. Such GABHS-mediated immune activation might also occur through the involvement of GABHS superantigens, a mechanism currently under investigation. Very preliminary evidence supporting a superantigen-mediated mechanism comes from Kawikova et al. [11], who showed that a specific subset of T cells (V-beta18-positive), which recognize a specific type of GABHS superantigen, is abnormally reduced in TS children, possibly as a consequence of functional exhaustion following prolonged superantigenic exposure. Finally, dysgammaglobulinemia might make TS patients more susceptible to GABHS replication and colonization, thus enhancing a pathogenic vicious circle.

Might the hyper-dopaminergic state hypothesized to occur in TS be also responsible for the abnormal immune response in these patients? Importantly, dopamine modulates immune cells in autocrine or paracrine fashion, and lymphocytes express dopamine receptors on their surface [62]. Other neurological and psychiatric conditions associated with a dysfunction of dopaminergic circuits, such as Parkinson's disease and schizophrenia, also present with altered immune function. Abnormal dopaminergic modulation might be one cause of the generalized lymphocyte activation and the disinhibited cytokine secretion detected in TS patients. In addition, Tregs are inhibited by dopamine through activation of D1/D5 dopamine receptors, which have been recently found to be down-regulated in peripheral lymphocytes of TS patients [63]. Finally, lymphocytes may be modulated also by other neurotransmitters including glutamate, acetylcholine and serotonin, which might also be involved in the pathophysiology of TS and tic disorders in general.

As a final question for future research, it is interesting to hypothesize whether the altered function of immune cells might contribute to abnormal brain maturation, and consequently facilitate the manifestation of tics and associated dysfunctional behaviours. Although this hypothesis has been explored in other neurodevelopmental disorders, primarily in autism, there is no evidence at present to support this complex neural-immune interplay in tic disorders. In any case, immune molecules are known to modulate neurogenesis, neuronal migration and synaptogenesis, as well as contribute to activity-dependent synaptic plasticity [64]. Future progress in the development of more valid animal models replicating the abnormal trajectories of maturation of fronto-subcortical and cortico-cortical networks involved in tic generation, as well as larger availability of pathological brain specimens will be necessary to explore this fascinating aspect of TS pathogenesis.
